# Supplementary material for: Voltage Controlled Magnetic Skyrmion Motion for Racetrack Memory
Source: Sci Rep. 2016 Mar 15;6:23164. doi: 10.1038/srep23164 (PMC4791601; doi:10.1038/srep23164)
Supplement: Supplementary Information [file srep23164-s1.pdf]

Voltage Controlled Magnetic Skyrmion Motion for Racetrack Memory

Wang Kang[1,2,3], Yangqi Huang[1,2], Chentian Zheng[1], Weifeng Lv[3], Na Lei[1], Youguang Zhang[1,2], Xichao Zhang[4], Yan Zhou[4,6†], and Weisheng Zhao[1,2,5,*]

[1] *Fert Beijing Institute, Beihang University, Beijing, China*
[2] *School of Electronic and Information Engineering, Beihang University, Beijing, China*
[3] *School of Computer Science and Engineering, Beihang University, Beijing, China*
[4] *Department of Physics, University of Hong Kong, Hong Kong, China*
[5] *Institut d'Electronique Fondamentale (IEF), Univ. Paris-Sud, CNRS, Orsay, France*
[6] *School of Electronic Science and Engineering, Nanjing University, Nanjing, China*

*E-mail: weisheng.zhao@buaa.edu.cn

†E-mail: yanzhou@hku.hk

SUPPLEMENTARY INFORMATION

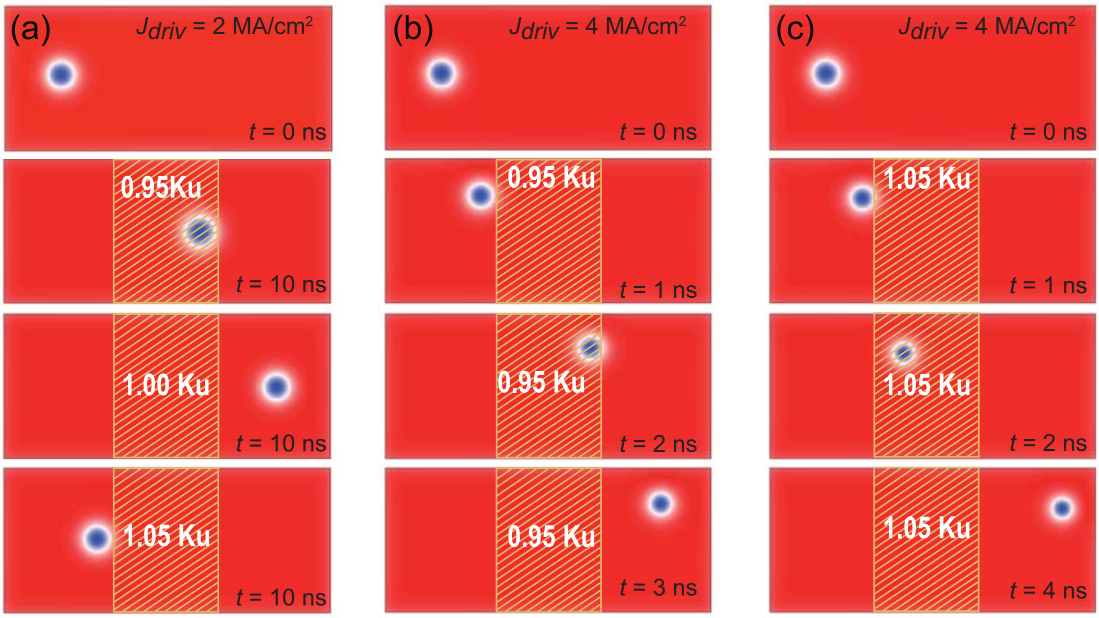

Figure S1. The top-view of the nanotracks (with baseline $K_u = 0.8\ \mathrm{MJ/m^3}$ and width of 180 nm) under different driving current densities as well as the applied voltages (or energy barriers) of the VCMA gate. (a) $j_{driv} = 2\ \mathrm{MA/cm^2}$, $K_{uv} = 0.95\ K_u$, $1.00\ K_u$ or $1.05\ K_u$; (b) $j_{driv} = 4\ \mathrm{MA/cm^2}$ and $K_{uv} = 0.95\ K_u$; (c) $j_{driv} = 4\ \mathrm{MA/cm^2}$ and $K_{uv} = 1.05\ K_u$.

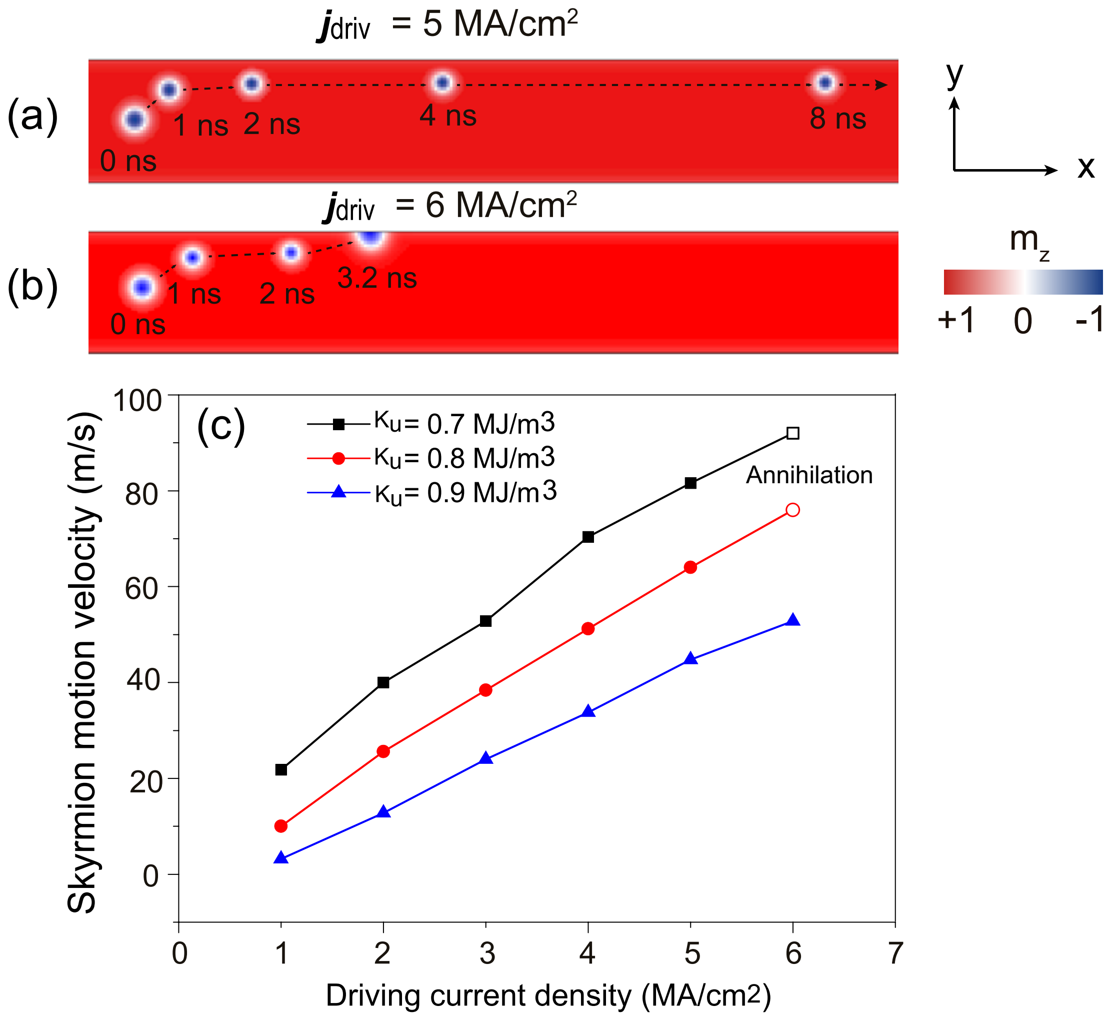

Figure S2. (a) Trajectory of a skyrmion driven by a current with density of $j_{driv} = 5\ \mathrm{MA/cm^2}$ and (b) with density of $j_{driv} = 6\ \mathrm{MA/cm^2}$. For the case of $j_{driv} = 5\ \mathrm{MA/cm^2}$, the skyrmion moves steadily along the nanotrack. However, for $j_{driv} = 5\ \mathrm{MA/cm^2}$, the skyrmion reaches the nanotrack edge and be annihilated. (c) The skyrmion motion (longitudinal) velocity with respect to the driving current density for different magnetic anisotropy (K_u) values. Here the hollow square and circle denote the cases that the skyrmions are annihilated.

Supplementary Movie Captions

Supplementary Movie 1. The trajectory of a skyrmion in the VCMA-gated nanotrack, in which the VCMA gate is controlled by using on/off positive voltage pulse (corresponding to Figure 3a of the main text.

Supplementary Movie 2. The trajectory of a skyrmion in the VCMA-gated nanotrack, in which the VCMA gate is controlled by using on/off negative voltage pulse (corresponding to Figure 3b of the main text.

Supplementary Movie 3. The trajectory of a skyrmion in the VCMA-gated nanotrack, in which the VCMA gate is controlled by using a constant positive voltage (corresponding to Figure 4a of the main text.

Supplementary Movie 4. The trajectory of a skyrmion in the VCMA-gated nanotrack, in which the VCMA gate is controlled by using a constant negative voltage (corresponding to Figure 4b of the main text.

Supplementary Movie 5. The trajectory of a skyrmion driven by spin current pulses with density of $j_{driv} = 6\ \mathrm{MA/cm^2}$ and pulse width of 2 ns, corresponding to Figure 8a of the main text. The skyrmion moves steadily along the nanotrack.

Supplementary Movie 6. The trajectory of a skyrmion driven by spin current pulses with density of $j_{driv} = 6\ \mathrm{MA/cm^2}$ and pulse width of 3 ns, corresponding to Figure 8b of the main text. The skyrmion reaches the nanotrack edge and is annihilated.
