# Supplementary figures and images for: Voltage Controlled Magnetic Skyrmion Motion for Racetrack Memory
Source: Sci Rep. 2016 Mar 15;6:23164. doi: 10.1038/srep23164 (PMC4791601; doi:10.1038/srep23164)

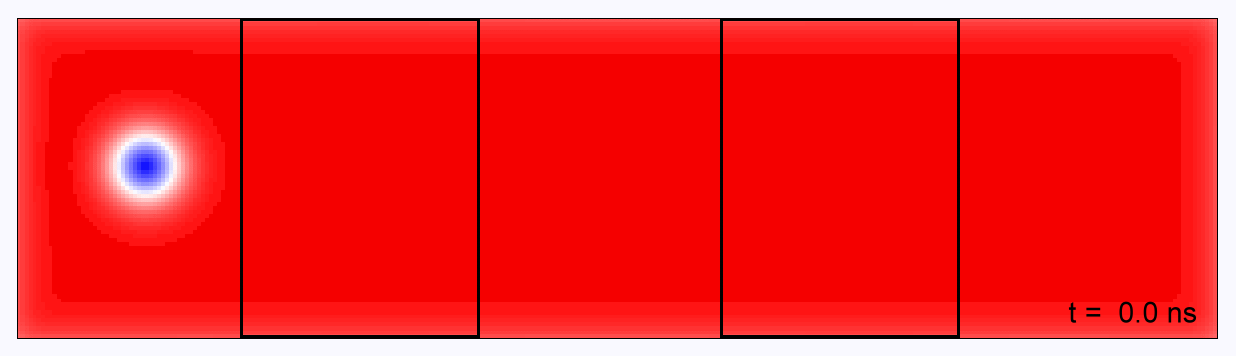

Supplement: Supplementary Movie 1 [file srep23164-s2.gif]

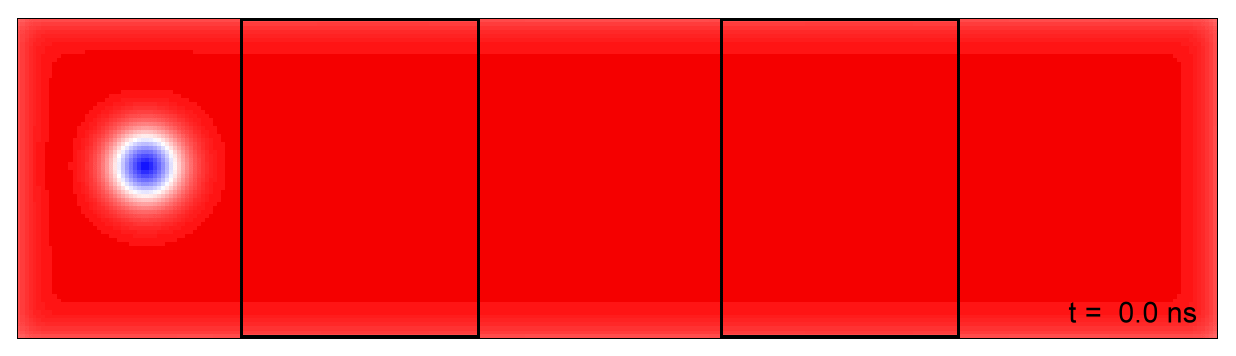

Supplement: Supplementary Movie 2 [file srep23164-s3.gif]

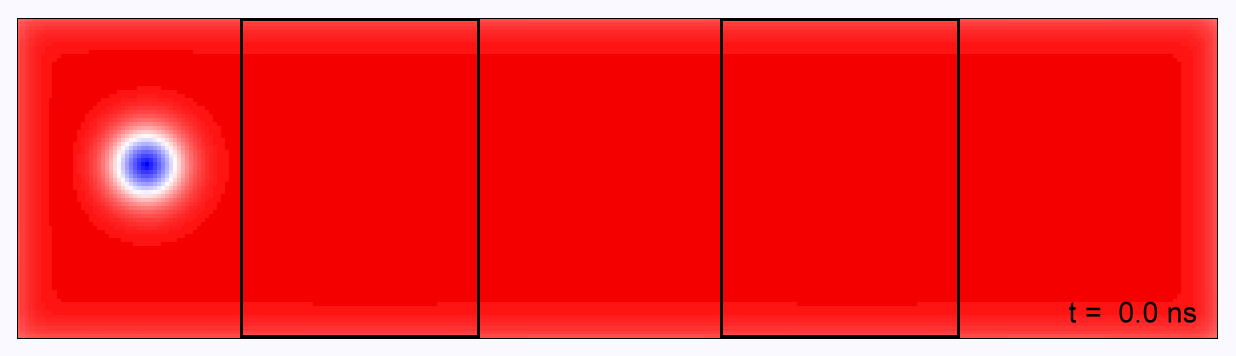

Supplement: Supplementary Movie 3 [file srep23164-s4.gif]

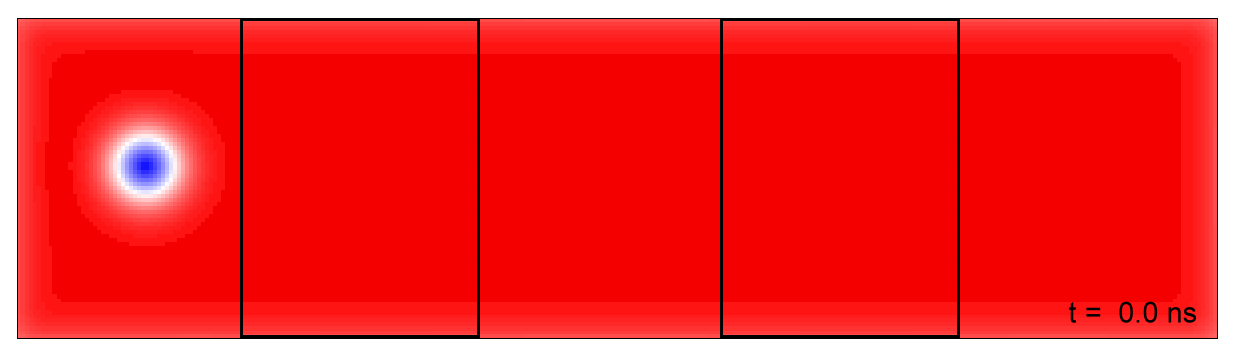

Supplement: Supplementary Movie 4 [file srep23164-s5.gif]

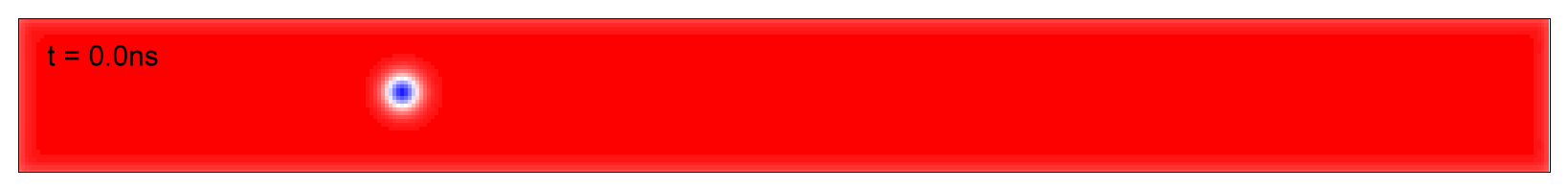

Supplement: Supplementary Movie 5 [file srep23164-s6.gif]

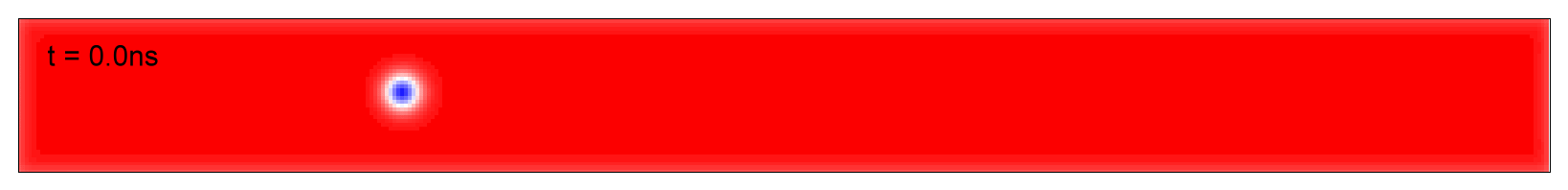

Supplement: Supplementary Movie 6 [file srep23164-s7.gif]
